# Mimicking the Oxygen-Evolving Center in Photosynthesis

**DOI:** 10.3389/fpls.2022.929532

**Published:** 2022-07-07

**Authors:** Yang Chen, Boran Xu, Ruoqing Yao, Changhui Chen, Chunxi Zhang

**Affiliations:** ^1^Laboratory of Photochemistry, Institute of Chemistry, Chinese Academy of Sciences, Beijing, China; ^2^University of Chinese Academy of Sciences, Beijing, China

**Keywords:** photosystem II, oxygen-evolving center, Mn_4_CaO_4_-cluster, artificial photosynthesis, water-splitting reaction

## Abstract

The oxygen-evolving center (OEC) in photosystem II (PSII) of oxygenic photosynthetic organisms is a unique heterometallic-oxide Mn_4_CaO_5_-cluster that catalyzes water splitting into electrons, protons, and molecular oxygen through a five-state cycle (S_n_, *n* = 0 ~ 4). It serves as the blueprint for the developing of the man-made water-splitting catalysts to generate solar fuel in artificial photosynthesis. Understanding the structure–function relationship of this natural catalyst is a great challenge and a long-standing issue, which is severely restricted by the lack of a precise chemical model for this heterometallic-oxide cluster. However, it is a great challenge for chemists to precisely mimic the OEC in a laboratory. Recently, significant advances have been achieved and a series of artificial Mn_4_XO_4_-clusters (X = Ca/Y/Gd) have been reported, which closely mimic both the geometric structure and the electronic structure, as well as the redox property of the OEC. These new advances provide a structurally well-defined molecular platform to study the structure–function relationship of the OEC and shed new light on the design of efficient catalysts for the water-splitting reaction in artificial photosynthesis.

## Introduction

**Figure 1:**
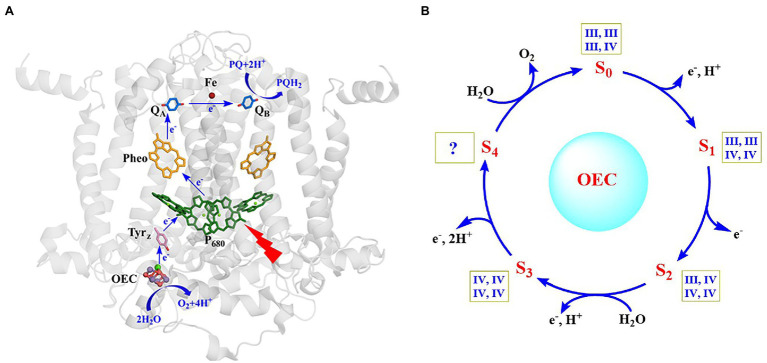
Structure of the reaction center and main cofactors involved in electron transfer in PSII (Umena et al., 2011) **(A)** and catalytic cycle of the OEC (Cox et al., 2020) **(B)**. The oxidation states of four Mn ions in each S-state are shown within square boxes.

Photosynthetic oxygen evolution is a unique function of oxygenic photosynthetic organisms, which takes place in photosystem II (PSII) of cyanobacteria, algae, and plants (Barber, 2009; Dau and Zaharieva, 2009; Cardona et al., 2012; Vinyard et al., 2013; Shen, 2015; Govindjee et al., 2017; Junge, 2019; Lubitz et al., 2019; Shevela et al., 2019; Blankenship, 2021). PSII is a multi-subunit membrane protein complex containing more than 20 subunits and hundreds of cofactors. The reaction center of PSII is shown in Figure 1A. Upon photo excitation, the primary electron donor (P_680_) donates one electron to the primary electron acceptor (Pheo) in a few picoseconds, producing the P_680_^+•^ and Pheo^-•^ at the donor side and acceptor side, respectively (Renger and Holzwarth, 2005; Rappaport and Diner, 2008; Cardona et al., 2012; Shevela et al., 2021). Pheo^-•^ then delivers the electron to the primary plastoquinone (Q_A_) and the secondary plastoquinone (Q_B_) in sequence *via* the non-heme iron at the acceptor side (Petrouleas and Crofts, 2005; Cardona et al., 2012), where one bicarbonate anion coordinated on the non-heme iron is highly required for the efficient electron transfer between Q_A_ and Q_B_ (Shevela et al., 2012). P_680_^+•^ with high redox potential (~1.25 V) abstracts one electron from the secondary electron donor (Tyr_Z_), forming a neutral radical (Tyr_Z_^•^) (Diner and Britt, 2005; Styring et al., 2012). The latter then drives the water-splitting reaction at the oxygen-evolving center (OEC) in milliseconds at the donor side (Tommos and Babcock, 1998; Zhang, 2007; Styring et al., 2012). The catalytic turnover of the OEC (Figure 1B) involves five different redox states (S_n_, *n* = 0 ~ 4) (Kok et al., 1970; Dau and Haumann, 2007; Cox et al., 2014a; Yano and Yachandra, 2014), in which the S_0_ state is the initial and most reduced state, the S_1_ state is the dark-stable state, the S_2_ and S_3_ states are metastable and decay eventually to the dark stable S_1_ state, whereas the S_4_ state is a transient state that releases molecular oxygen and regenerates the S_0_ state. This catalytic water-splitting reaction provides electrons and protons to, ultimately, produce the biomass or biofuel, and molecular oxygen to maintain the oxygenic atmosphere of our planet (Blankenship et al., 2011; Barber, 2020), which serves as the blueprint to develop efficient man-made catalysts for the water-splitting reaction in artificial photosynthesis.

Due to broad interests in fundamental research and potential applications in artificial photosynthesis (Herrero et al., 2008; Gust et al., 2010; Andreiadis et al., 2011; Concepcion et al., 2012; Faunce et al., 2013; Kärkäs et al., 2014; Hunter et al., 2016; Najafpour et al., 2016; El-Khouly et al., 2017; Nocera, 2017; Ye et al., 2019; Zhang and Sun, 2019b; Zhang and Reisner, 2020; Kondo et al., 2021), the structure and catalytic mechanism of the OEC have attracted extensive attention during the last century (Junge, 2019; Cox et al., 2020). However, revealing the principle of the OEC has been one of the great and persistent challenges and a long-standing issue in the research field of photosynthesis.

## Structure of the OEC

Extensive biochemistry and biophysics studies have been performed to reveal the properties of the OEC in different S-states during the last several decades (Yano and Yachandra, 2014; Junge, 2019). It has been well demonstrated that OEC is composed of four Mn ions and one calcium ion, in which the calcium can be replaced by strontium (Debus, 1992; Yocum, 2008). Based on spectroscopic studies of the X-ray absorption spectroscopy (XAS) (Yano and Yachandra, 2014) and electron paramagnetic resonance (EPR) (Peloquin and Britt, 2001; Krewald et al., 2015) measurements of different S-states OEC, the oxidation states of the four Mn ions were generally suggested to be S_0_ (III, III, III, IV), S_1_ (III, III, IV, IV), S_2_ (III, IV, IV, IV), and S_3_ (IV, IV, IV, IV), respectively. However, some groups (Zheng and Dismukes, 1996; Gatt et al., 2012; Pace et al., 2012; Petrie et al., 2020) suggested that the oxidation states of the four Mn ions could be S_0_ (II, III, III, III), S_1_ (III, III, III, III), S_2_ (III, III, III, IV), and S_3_ (III, III, IV, IV), respectively. These two different assignments of the four Mn ions are labeled as the “high-oxidation paradigm” and the “low oxidation paradigm,” respectively (Krewald et al., 2015; Pantazis, 2018). The former has generally been used by most researchers, yet the unambiguous chemical evidence for the assignment of the oxidation states of the four Mn ions remains elusive.

**Figure 2:**
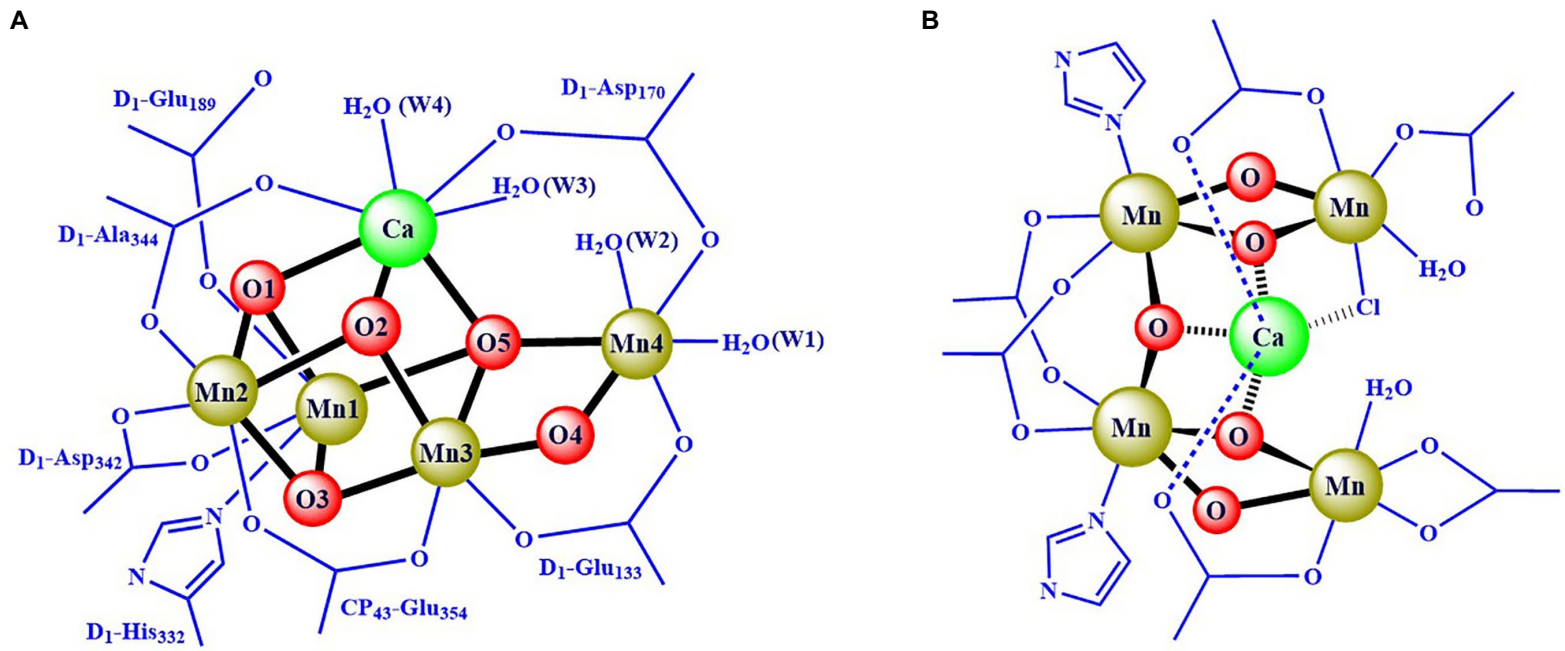
Scheme for the structure of the OEC (Umena et al., 2011) **(A)** and the theoretical model (Zhang et al. 1999) **(B)**.

The crystal structure information of the OEC has emerged since the beginning of this century (Zouni et al., 2001; Kamiya and Shen, 2003; Ferreira et al., 2004; Loll et al., 2005; Guskov et al., 2009; Tanaka et al., 2017; Graça et al., 2021; Kato et al., 2021). In 2001, Zouni et al. reported a crystal structure of PSII from a cyanobacterium at a resolution of 3.8 Å (Zouni et al., 2001). In 2004, Ferreira et al. (Ferreira et al., 2004) reported a resolution of 3.5 Å structure data of PSII and proposed that the OEC was a Mn_3_CaO_4_ cubane attached by a “dangler” Mn ion *via* one μ_4_-oxide bridge, forming a Mn_4_CaO_4_-cluster (Ferreira et al., 2004). In 2011, Umena et al. (Umena et al., 2011) reported the crystal structure of PSII at a resolution of 1.9 Å, which revealed the detailed structure of the OEC, as shown in Figure 2A. Here, the coordination ligands of the OEC are provided by six carboxylate groups from the amino acid residues of D_1_-Asp_170_, D_1_-Glu_189_, D_1_-Glu_333_, D_1_-Asp_342_, D_1_-Ala_344_, CP_43_-Glu_354_, one imidazole group from D_1_-His_332_, and four water molecules (two on calcium and two on dangler Mn, respectively). Further, an additional μ_2_-oxide bridge (O4) between Mn4 and Mn3 was observed (Umena et al., 2011), which is consistent with the proposal by Dau et al. (2008). The structure of the OEC, shown in Figure 2A, has been further confirmed by the X-ray free-electron laser (XFEL) data (Kupitz et al., 2014; Suga et al., 2015, 2017, 2019; Young et al., 2016; Kern et al., 2018; Ibrahim et al., 2020; Hussein et al., 2021) and the single-particle cryo-electron microscopy (Cryo-EM) (Wei et al., 2016; Kato et al., 2021; Xiao et al., 2021; Gisriel et al., 2022). The entire structure of the OEC is an asymmetric Mn_4_CaO_5_-cluster. In this cluster, calcium, a key component of the OEC, is located in the middle and connected to the four Mn ions through three oxide bridges and two carboxylate groups; this structural feature (see Figure 2B) is consistent with the proposal by Zhang et al. in 1999 (Zhang et al., 1999).

Notably, most crystallographic studies, in the past, were performed on the dark stable PSII sample, in which the OEC was generally in the S_1_ state. Recently, the structures of the OEC in other S-states have been reported (Suga et al., 2017, 2019; Kern et al., 2018; Ibrahim et al., 2020; Hussein et al., 2021; Li et al., 2021). Remarkably, one new oxygen atom (O_6_) coordinated to the Mn1 was observed in the S_3_ state. This new oxygen atom was suggested to serve as one of the substrates to form the O=O bond (Suga et al., 2017, 2019; Kern et al., 2018; Ibrahim et al., 2020). However, the existence of the new oxygen (O_6_) in the S_3_ state is still under debate. It was argued that both O_6_ and O_5_ in the reported S_3_ state OEC may belong to the same oxygen atom but in two possible positions (Petrie et al., 2020; Wang et al., 2021). Furthermore, there are some structural uncertainties due to the incoherent transition of the S-state of PSII samples (Askerka et al., 2015; Tanaka et al., 2017).

Compared with the structure revealed by the X-ray diffraction (XRD) (Ferreira et al., 2004; Loll et al., 2005; Umena et al., 2011; Tanaka et al., 2017), the structure of the OEC revealed by the XFEL (Kupitz et al., 2014; Suga et al., 2015, 2017, 2019; Young et al., 2016; Kern et al., 2018; Ibrahim et al., 2020; Hussein et al., 2021) has been considered to be more reliable due to the lack of significant radiation damage induced by the X-ray beam (Yano et al., 2005; Grabolle et al., 2006). However, as yet, there is consensus for the atomic positions of the S_1_ state OEC, as revealed by XFEL, certainly not for all structures with the results from EXAFS spectroscopy studies on the active sample used (Davis and Pushkar, 2015; Askerka et al., 2017). To check if those reported structure data were directly correlated with the native structures of the OEC in different S-states, we have carried out bond valence sum (BVS) calculations on these XFEL’s structures reported recently (Chen et al., 2019; Li et al., 2020b). We note that BVS calculation has been widely used to evaluate the oxidation valences of atoms in coordination complexes and in metalloenzymes (Brown, 2009). Table 1 lists the results of the BVS calculation on the XFEL’s structures of different S-states of the OEC reported recently. Surprisingly, we see that the oxidation states of the four Mn ions revealed by BVS calculations are significantly lower than that suggested by the spectroscopic studies (Peloquin and Britt, 2001; Dau and Haumann, 2007; Yano and Yachandra, 2014; Krewald et al., 2015) (Figure 1B), indicating that the reduction of the Mn ions with high valences would take place during the structural determination by XFEL (Yano et al., 2005; Grabolle et al., 2006; Amin et al., 2016). If so, one would expect that those reported structure data of the OEC would be different from the native structure. This opinion is consistent with the suggestion that structural modifications of the OEC induced by XFEL may take place and the position of the oxide bridge (eg., O_5_) could be significantly disturbed by XFEL (Amin et al., 2016). Therefore, the precise structure of the OEC in different S-states remains elusive.

**Table 1:** Bond valence sum (BVS) calculations on the structure of the OEC revealed by X-ray free-electron laser (XFEL) method at different resolutions in different S-states.

	S_1_ (1.95 Å) 4UB6	S_1_ (2.05 Å) 6DHE	S_2_ (2.15 Å) 6JLK	S_2_ (2.08 Å) 6DHF	S_3_ (2.07 Å) 6DHO	S_3_ (2.15 Å) 6JLL
Mn1	3.075 (III)	3.101(III)	3.000(III)	3.232(III)	3.901(IV)	4.203(IV)
Mn2	3.237 (III)	4.277(IV)	3.735(IV)	4.316(IV)	4.193(IV)	3.792(IV)
Mn3	2.980 (III)	3.670(IV)	2.946(III)	3.784(IV)	3.232(III)	3.181(III)
Mn4	2.318 (II)	2.870(III)	2.623(III)	3.139(III)	2.932(III)	2.551(II)

Roman numerals in parentheses indicate the assignment of the oxidation state of Mn ion based on BVS calculation. All atomic coordinates used for BVS calculation were taken from the first monomer of PS II in the crystal structure data with PDB-IDs: 4UB6 (Suga et al., 2015), 6DHE (Kern et al., 2018), 6DHF (Kern et al., 2018), 6DHO (Kern et al., 2018), 6JLK (Suga et al., 2019), and 6JLL (Suga et al., 2019), respectively.

## Catalytic Mechanism of the OEC

**Figure 3:**
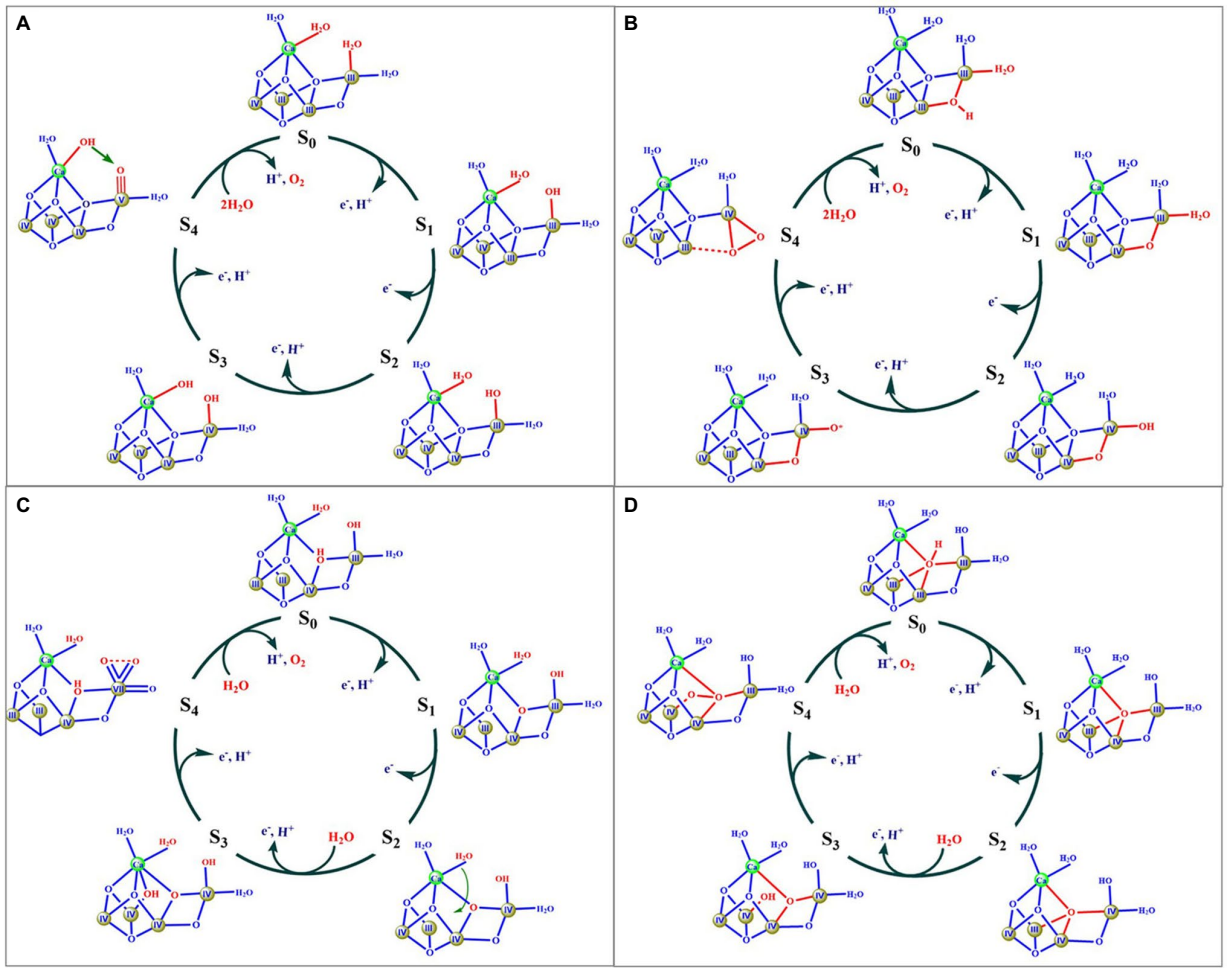
Four typical mechanism models suggested in literatures. Components of the OEC involved in O=O bond formation are marked by red color. Roman numbers indicate the oxidation state of the Mn ion.

Based on biochemical, biophysical, and theoretical investigations, various hypotheses for the catalytic mechanisms of the O=O bond formation have been suggested by many researchers (Siegbahn, 2013; Askerka et al., 2017; Barber, 2017; Wang et al., 2017; Corry and O’Malley, 2018; Kawashima et al., 2018; Kern et al., 2018; Pushkar et al., 2018; Yamaguchi et al., 2018; Britt and Marchiori, 2019; Suga et al., 2019; Zhang and Sun, 2019a; Marchiori et al., 2020; Capone et al., 2021). Figure 3 shows four typical proposals.

Figure 3A shows a possible mechanism proposed by Barber (Barber, 2017), in which two water molecules (W2 and W3) serve as substrates to form the O=O bond. The key feature of this mechanism is that the O=O bond is formed by a nucleophilic attack of a calcium ligated hydroxyl group onto an electrophilic oxo group of Mn^V^ ≡ O or Mn^IV^-O• derived from the deprotonation of the second substrate water molecule. Similar proposals had also been suggested by others (Pecoraro et al., 1998; Vrettos et al., 2001; Chen et al., 2015; Vinyard et al., 2015). However, these proposals were not supported by recent theoretical calculations reported by Siegbahn group (Siegbahn, 2017). The second model (Figure 3B) was suggested by Ishikita group (Kawashima et al., 2018), in which the μ_2_-oxide bridge (O4) and one water molecule (W1) serve as two oxygen sources to form the O=O bond. The key feature of this proposal is that the O=O bond is formed through the coupling of the O4 oxide bridge and a Mn^IV^-O• oxyl radical. However, the oxidation states of (III, IV, IV, IV) for the four Mn ions in the S_3_ state were not consistent with the widely accepted oxidation states of (IV, IV, IV, IV) (Peloquin and Britt, 2001; Dau and Haumann, 2008; Yano and Yachandra, 2014; Krewald et al., 2015). The third model (Figure 3C) was proposed by Sun group (Zhang and Sun, 2019a), in which one Mn^VII^ ion was suggested to be involved in the S_4_ state. This mechanism has been recently evaluated by a computational study that shows that the formation of the Mn^VII^ requires a much higher barrier for forming O_2_ than the earlier proposals with four Mn^IV^ atoms (Li et al., 2020a). The fourth model, originally proposed by Siegbahn (Siegbahn, 2013), and then other groups (Pecoraro et al., 1998; Cox et al., 2020), is where the μ_4_-oxide bridge (O5) and the newly inserted water (O_6_) are considered to serve as the oxygen source for the O=O bond (Figure 3D). This proposal has been widely used to explain the observation of the crystallographic data and a large number of spectroscopic observations (Cox et al., 2014b, 2020; Kern et al., 2018; Britt and Marchiori, 2019; Suga et al., 2019). According to this mechanism, the release of O_2_ from the S_4_ state would result in the formation of four unsaturated metal ions, namely three 5-coordinated manganese (i.e., Mn1, Mn3, and Mn4) and one 6-coordinated calcium, which would certainly require a much higher activation energy (Zhang and Kuang, 2018). Thus, one would expect that the molecular oxygen release could be the rate-limiting step during the catalytic cycle; however, this is inconsistent with the fast O_2_ release observed in PSII (Haumann et al., 2005; Davis et al., 2018).

As mentioned above, although various hypotheses for the mechanism of the water-splitting reaction of the OEC have been proposed (Siegbahn, 2013; Askerka et al., 2017; Barber, 2017; Wang et al., 2017; Corry and O’Malley, 2018; Kawashima et al., 2018; Kern et al., 2018; Pushkar et al., 2018; Yamaguchi et al., 2018; Britt and Marchiori, 2019; Suga et al., 2019; Zhang and Sun, 2019a; Marchiori et al., 2020; Capone et al., 2021), the detailed mechanism remains an open question mainly due to the lack of the unambiguous experimental evidence for the O=O bond formation and the precise geometric structure and electronic structure of the OEC in different S-states (Chen and Zhang, 2021).

## Mimicking the OEC

**Figure 4:**
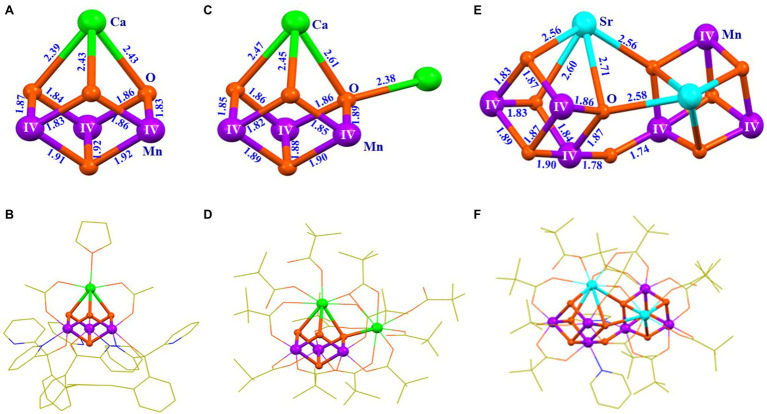
Structures of three artificial complexes containing Mn_3_XO_4_ cubane (X = Ca/Sr). **(A)** Core of the Mn_3_CaO_4_-complex (**1**); **(B)** Structure of the Mn_3_CaO_4_-complex (**1**) (Kanady et al., 2011); **(C)** Core structure of the Mn_3_Ca_2_O_4_-complex (**2**); **(D)** Structure of the Mn_3_Ca_2_O_4_-complex (**2**) (Mukherjee et al., 2012); **(E)** Core of the (Mn_3_SrO_4_)_2_O-complex (**3**); **(F)** Structure of the (Mn_3_SrO_4_)_2_O-complex (**3**) (Chen et al., 2014). Distances are given in Å units; Mn, Ca, Sr., O, N, and C are shown in purple, green, cyan, orange, blue, and yellow, respectively. For clarity, all hydrogen atoms are not shown.

To facilitate the understanding of the structure and properties of the OEC, as well as for developing efficient water-splitting catalysts, many research groups, during the last three decades, have attempted to synthesize the OEC (Wieghardt, 1989; Limburg et al., 1999; Mukhopadhyay et al., 2004; Mullins and Pecoraro, 2008; Dismukes et al., 2009; Gerey et al., 2016; Zhang and Sun, 2019b; Li et al., 2020b; Chen et al., 2021; Ezhov et al., 2021). It is a great challenge and a long-standing issue for chemists to synthesize the OEC in the laboratory (Zhang, 2015; Li et al., 2020b). During the last two decades, numerous multi-manganese complexes have been synthesized (Wieghardt, 1989; Limburg et al., 1999; Mukhopadhyay et al., 2004; Mullins and Pecoraro, 2008; Dismukes et al., 2009; Gerey et al., 2016; Chang et al., 2017). Significant advances for the mimicking of the OEC have emerged since 2011 (Tsui et al., 2013a; Chen et al., 2017; Paul et al., 2017). Further, Agapie group (Kanady et al., 2011) reported an artificial Mn^IV^_3_CaO_4_-complex (**1**) using a multi-pyridylalkoxide ligand (Figures 4A,B). In addition, a series of analogs or derivatives of the cluster have been reported, by using a similar ligand (Tsui and Agapie, 2013; Kanady et al., 2014; Lin et al., 2015; Lionetti et al., 2019). In 2012, Christou group reported a Mn^IV^_3_Ca_2_O_4_-complex (**2**) with one Ca^2+^ attached to the Mn_3_CaO_4_ cubane (Mukherjee et al., 2012) (Figures 4C,D). A similar Mn_3_Ca_2_O_4_-complex was also isolated as a by-product during the synthesis of Mn_4_CaO_4_-cluster (Chen et al., 2022). Here, the peripheral ligands of the Mn_3_Ca_2_O_4_-cluster are provided by pivalic anions or neutral pivalic acid, which resembles to that of the OEC in PSII (Umena et al., 2011). In 2014, Zhang group (Chen et al., 2014) reported an artificial (Mn^IV^_3_SrO_4_)_2_O-complex (**3**) that contains both the heterometallic-oxide Mn_3_SrO_4_ cubane and all three types of oxide bridges (μ_2_-oxide, μ_3_-oxide, and μ_4_-oxide), as seen in the Sr^2+^-containing OEC (Koua et al., 2013) (Figures 4E,F).

**Figure 5:**
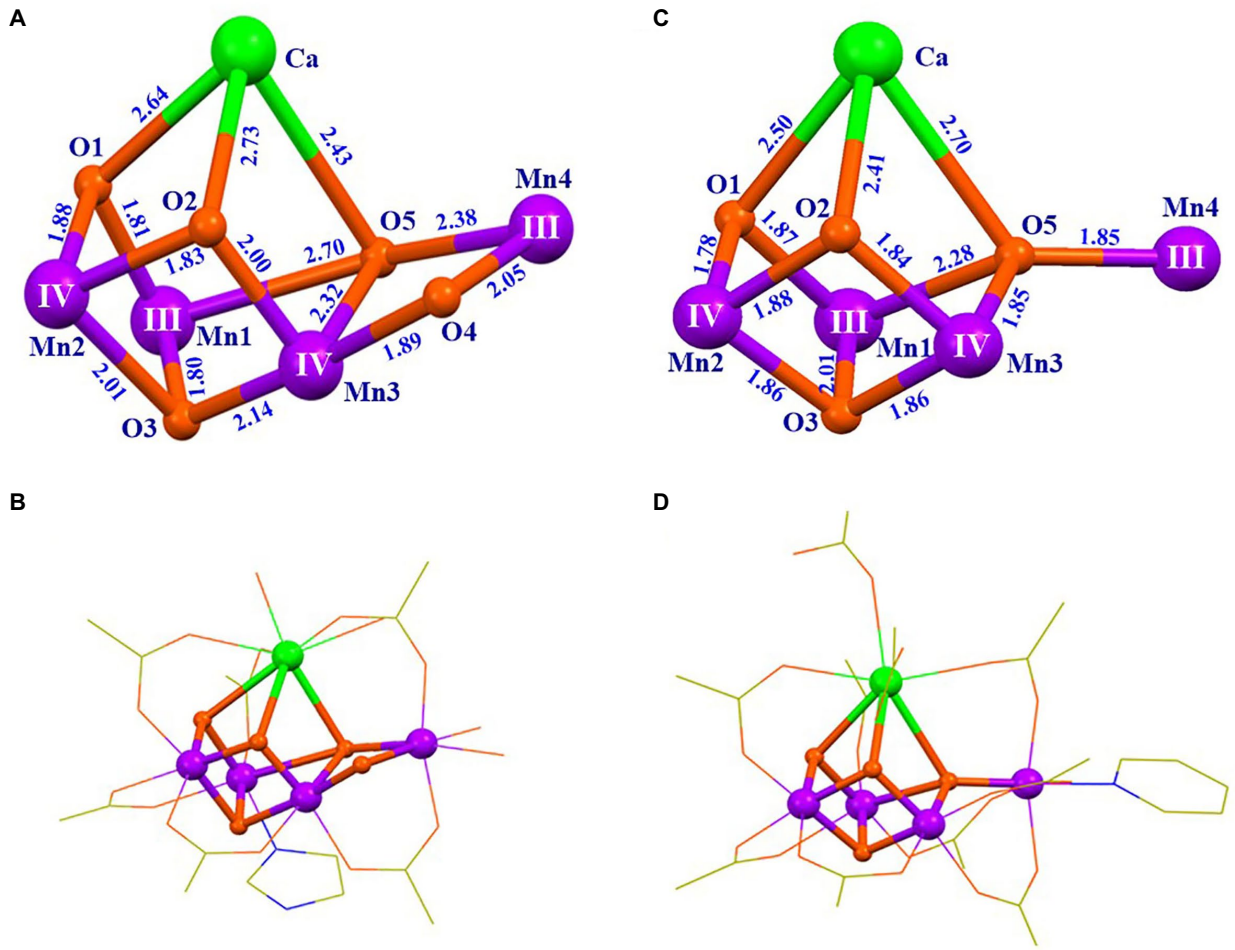
Structural comparison of the OEC (Suga et al., 2015) and the artificial Mn_4_CaO_4_-complex (**4**) (Zhang et al., 2015). **(A)** Core of the OEC; **(B)** structure of the OEC; **(C)** core of **4**; **(D)** structure of **4**. The data for the OEC is taken from the first monomer in the crystal structure data of PSII with the Protein Data Bank code 4UB6. For clarity, the methyl groups and the hydrogen atoms are not shown. All other illustrations are the same as those in Figure 4.

In 2015, Zhang group reported an artificial Mn_4_CaO_4_-complex (**4**; Figures 5C,D) that was prepared through a two-step procedure (Zhang et al., 2015). The first step was to synthesize a precursor through a reaction of Ca(CH_3_CO_2_)_2_•H_2_O, Mn(CH_3_CO_2_)_2_•(H_2_O)_4_, ^n^Bu_4_NMnO_4_ (^n^Bu = n-butyl), and ^t^BuCO_2_H (^t^Bu = tert-butyl; molar ratio of 1: 1: 4: 40) in boiling acetonitrile. The second step was to treat the precursor with 2% pyridine in ethyl acetate, leading to the formation of the final product, [Mn_4_CaO_4_(^t^BuCO_2_)_8_(^t^BuCO_2_H)_2_(C_5_H_5_N)] (**4**). This Mn_4_CaO_4_-complex contains a Mn_3_CaO_4_ cubane attached by a dangler Mn ion *via* one μ_4_-oxide bridge, forming an asymmetric Mn_4_CaO_4_-cluster. Its peripheral environment is provided by eight ^t^BuCO_2_^−^ anions and three neutral ligands on Ca and Mn4 (two pivalic acid molecules and one pyridine, respectively), which is remarkably similar to that in the OEC. BVS calculation confirms that the oxidation states of the four Mn ions are in (III, III, IV, IV).

**Figure 6:**
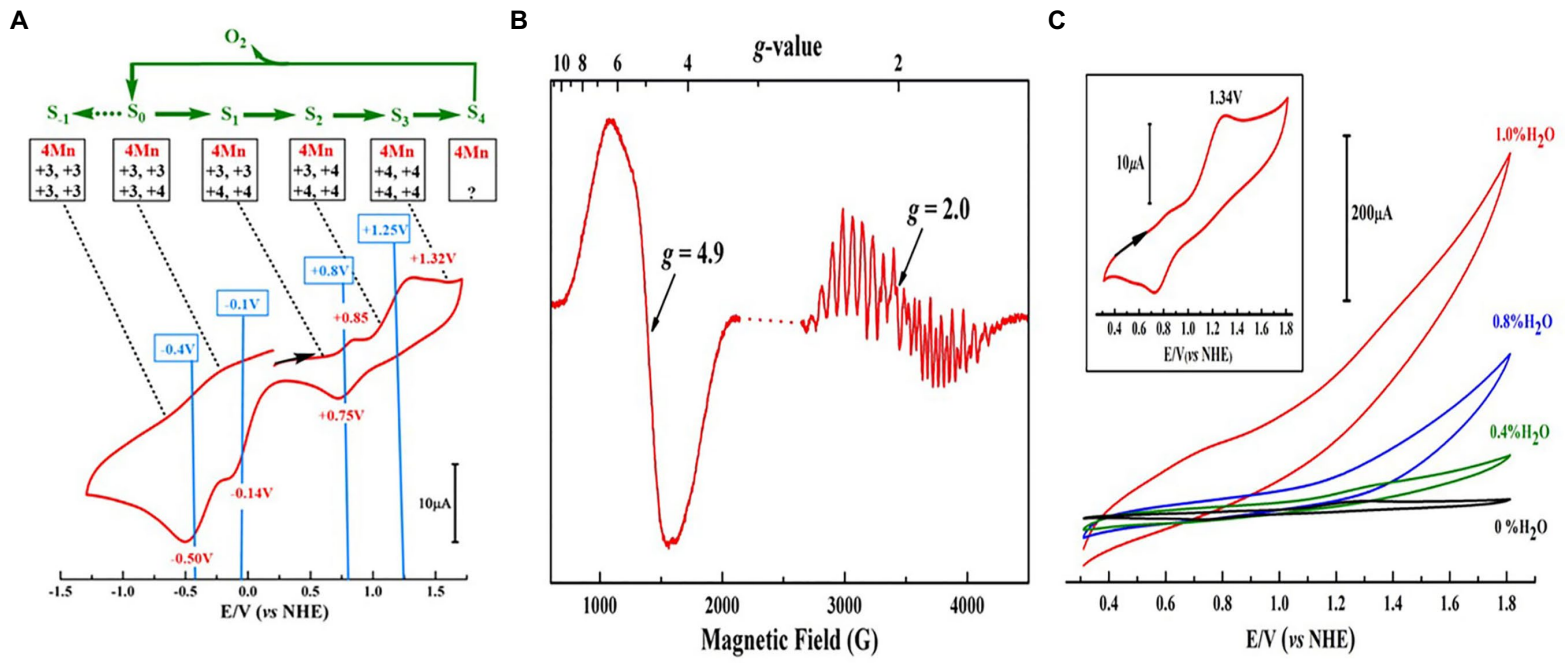
Redox properties, EPR, and catalytic activity measurements of Mn_4_CaO_4_-complex (**4**) (Zhang et al., 2015). **(A)** Cyclic voltammogram (CV) measurement of **4** in dichloroethane; **(B)** EPR spectrum for the one-electron oxidized **4**; **(C)** activity measurements of **4** in acetonitrile with different amounts of H_2_O. The inset in **C** shows the CV of **4** without H_2_O on a different scale.

The artificial Mn_4_CaO_4_-cluster (**4**) has a [Mn^III^_2_Mn^IV^_2_]/[Mn^III^Mn^IV^_3_] redox couple of ~0.8 V (vs. normal hydrogen electrode, NHE), as shown in the cyclic voltammogram (CV) (Figure 6A), which is essentially the same as the estimated value (~ 0.8 V) for the S_1_ → S_2_ transition of the OEC (Vass and Styring, 1991; Dau and Zaharieva, 2009; Mandal et al., 2020). The oxidized Mn_4_CaO_4_-cluster displays two distinct electron paramagnetic resonance (EPR) signals (*g* = 4.9 and *g* = 2.0) (Figure 6B), which are similar to the *g* ≈ 4 and *g* = 2.0 EPR signals observed in the S_2_ state OEC (Peloquin and Britt, 2001; Pantazis et al., 2012). Furthermore, the artificial Mn_4_CaO_4_-cluster can catalyze the water-splitting reaction on the electron surface in the presence of a small amount of water in acetonitrile (Figure 6C).

The artificial Mn_4_CaO_4_-cluster (**4**) is the closest mimic of the OEC up to now, which resembles not only in the structure of the metal-oxide core and the peripheral ligands, but also in the redox potential and the catalytic function of the OEC. Considering the high similarity between the artificial Mn_4_CaO_4_-cluster and the OEC, we speculate that oxidation states (III, III, IV, IV) of the four Mn ions in this artificial cluster provide unambiguous chemical evidence to support the assignment of oxidation states of (III, III, IV, IV) for the four Mn ions in the S_1_ state OEC in PSII.

## Synthesizing Mechanism of the Mn_4_CaO_4_-Cluster

**Figure 7:**
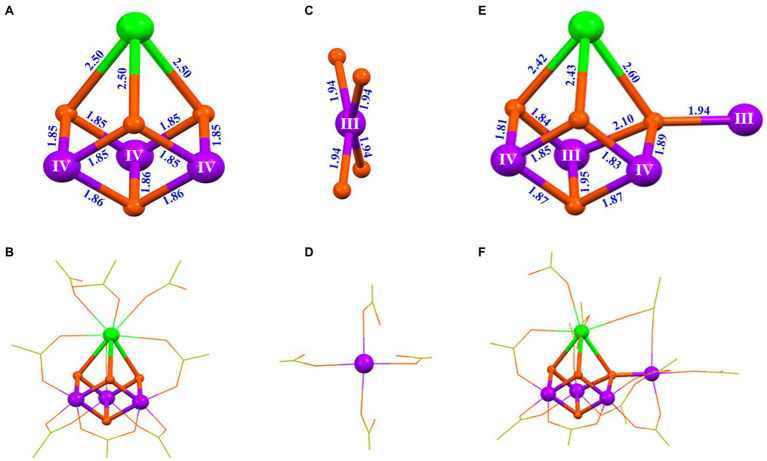
Structures of three intermediates for the synthesis of the artificial Mn_4_CaO_4_-cluster (Chen et al., 2022). **(A)** Core of [Mn_3_CaO_4_(tBuCO_2_)_6_(tBuCO_2_H)_3_] (**5**); **(B)** structure of **5**; **(C)** Mn center in [^n^Bu_4_NMn(^t^BuCO_2_)_4_] (**6**); **(D)** structure of the [Mn(^t^BuCO_2_)_4_]^−^ anion in **6**; **(E)** core structure of [Mn_4_CaO_4_(^t^BuCO_2_)_8_(^t^BuCO_2_H)_3_] (**7**); **(F)** structure of **7**. All other illustrations are the same as those in Figure 5.

The mechanism of the synthesis of the artificial Mn_4_CaO_4_-cluster has been recently studied by characterizing the intermediate species during the synthesis of the Mn_4_CaO_4_-complex (**4**) (Chen et al., 2022). By using the high-resolution electrospray ionization (HR-ESI) mass spectroscopy, we have characterized the precursor of the Mn_4_CaO_4_-cluster and observed five key fragments with m/z^−^ values at 1233.235, 1218.259, 875.118, 358.120, and 343.143 assigned to the [Mn_4_CaO_4_(^t^BuCO_2_)_9_]^−^, [Mn_3_Ca_2_O_4_(^t^BuCO_2_)_9_]^−^, [Mn_3_CaO_4_(^t^BuCO_2_)_6_]^−^, [Mn(^t^BuCO_2_)_3_]^−^, and [Ca(^t^BuCO_2_)_3_]^−^, respectively (Chen et al., 2022). More importantly, after extensive experimentation, three key intermediates, [Mn_3_CaO_4_(^t^BuCO_2_)_6_(^t^BuCO_2_H)_3_] (**5**), [^n^Bu_4_NMn(^t^BuCO_2_)_4_] (**6**), and [Mn_4_CaO_4_(^t^BuCO_2_)_8_(^t^BuCO_2_H)_3_] (**7**), were successfully crystallized. The structures of these intermediates (**5**–**7**) are shown in Figure 7.

**Figure 8:**
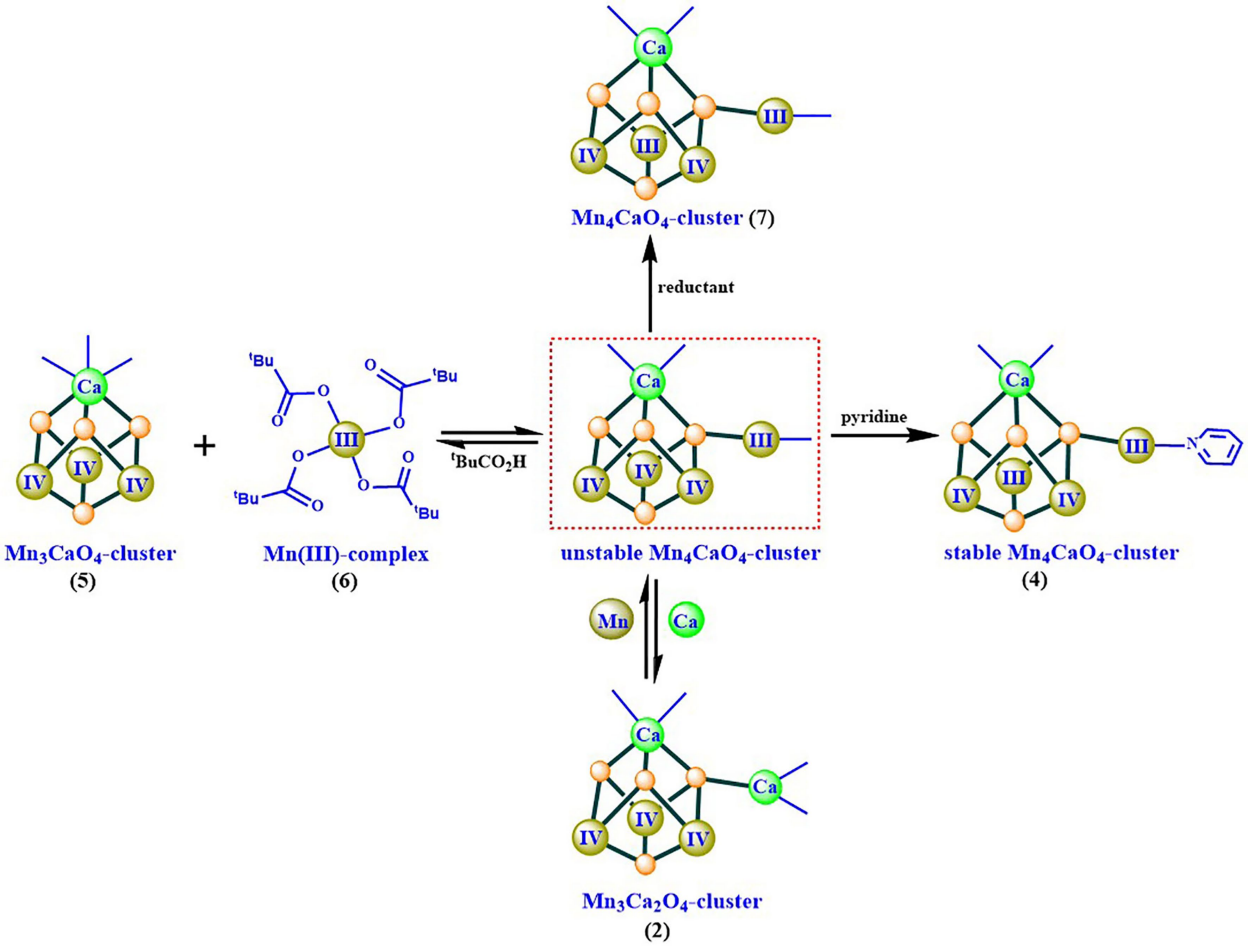
Possible synthesizing mechanism of the artificial Mn_4_CaO_4_-cluster (**4**) (Chen et al., 2022).

Based on the isolation and characterization of these intermediates for the synthesis of the Mn_4_CaO_4_-cluster, we suggest that the Mn_4_CaO_4_-cluster could be formed through a reaction between a thermodynamically stable Mn_3_CaO_4_-cluster and an unusual four-coordinated Mn^III^ ion (Figure 8). The freshly formed Mn_4_CaO_4_-cluster (**7**) with carboxylate groups only is unstable, but it can be significantly stabilized by binding an organic base (e.g., pyridine) on the “dangler” Mn ion. Furthermore, we have found that the dangler Mn ion is flexible and can be replaced by calcium under weak acid conditions, giving rise to the Mn_3_Ca_2_O_4_-cluster (**2**) as shown in Figures 4C,D.

Considering the high similarity between the artificial Mn_4_CaO_4_-cluster and the OEC (Figure 5), we speculate that the synthesizing mechanism (Figure 8), described above, could provide chemical insights into the assembly of the OEC. In the biological system, both the assembly and the disassembly of the OEC frequently take place under physiological conditions. The disassembly of the OEC takes place after the photodamage and degradation of the D_1_ protein of PSII under high light flux. To achieve the water-splitting capability, the newly functional OEC must be properly assembled after the repairing of the D_1_ protein of PSII (Barber and Andersson, 1992; Dasgupta et al., 2008). In PSII, the early steps of the assembly of the OEC involving two Mn and one Ca ions have been studied for more than 50 years (Cheniae and Martin, 1971; Dasgupta et al., 2008; Bao and Burnap, 2016; Murray et al., 2020); on the other hand, the assembly of the third and the fourth Mn ions in OEC is fully unknown (Bao and Burnap, 2016; Avramov et al., 2020). Considering the observed thermodynamical stability of the fully carboxylic ligand coordinated Mn_3_CaO_4_-cluster (**5**) observed (Chen et al., 2022), we propose that a similar Mn_3_CaO_4_-cluster could be present during the synthesis of the OEC in PSII. If it was the case, a mono-nuclear Mn ion (similar to that in **6**) would be necessary to be incorporated into the Mn_3_CaO_4_-cluster, followed by structural rearrangements to form the intact OEC, as has been suggested recently (Gisriel et al., 2020; Sato et al., 2021).

## Ligands Substituted OEC’s Mimics

**Figure 9:**
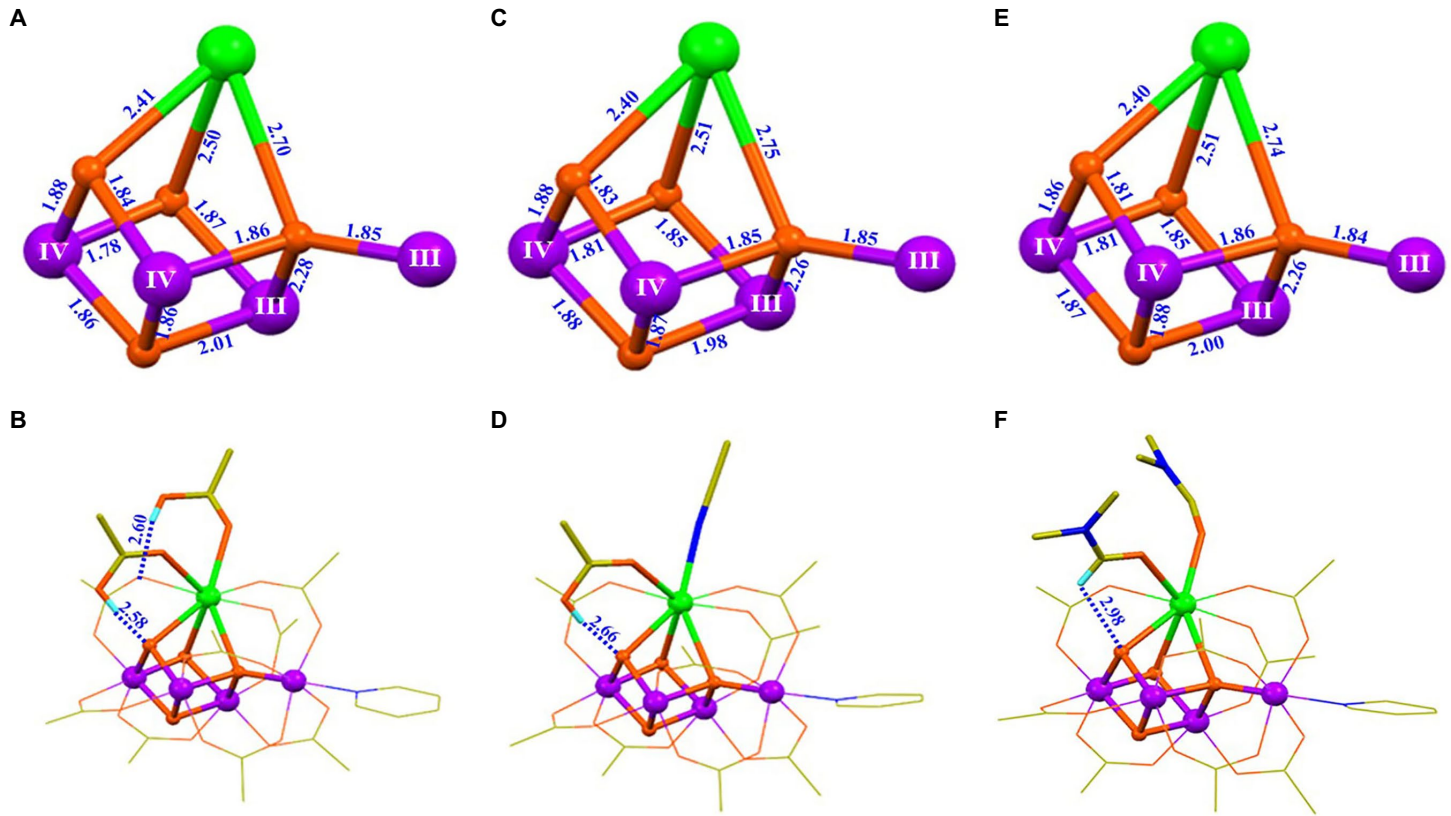
Structural comparison of three artificial Mn_4_CaO_4_-complexes (**4**, **8**, **9**) (Zhang et al., 2015; Chen et al., 2019). **(A)** Core of **4**; **(B)** structure of **4**; **(C)** core of **8**; **(D)** structure of **8**; **(E)** core of **9**; **(F)** structure of **9**. All illustrations are the same as those in Figure 5.

In order to improve the stability of the artificial Mn_4_CaO_4_-cluster, we have optimized its peripheral environment by replacing the two pivalic acid molecules on the calcium with organic solvent molecules (Chen et al., 2019). Structures of two new Mn_4_CaO_4_-complexes, [Mn_4_CaO_4_(^t^BuCO_2_)_8_(Py)(^t^BuCO_2_H) (CH_3_CN)] (**8**) and [Mn_4_CaO_4_(^t^BuCO_2_)_8_(Py)(DMF)_2_] (**9**) are shown in Figure 9. Interestingly, we have found that the change of these ligands on calcium does not affect neither the Mn_4_CaO_4_ core nor the oxidation states of the four Mn ions, as shown in Figure 9. This observation demonstrates that both the geometric structure and the electronic structure of the artificial Mn_4_CaO_4_-cluster are relatively stable, which provides chemical insights into the reason why the oxygenic photosynthetic organisms have selected the Mn_4_CaO_4_-cluster as the key structural unit to build the OEC in natural photosynthesis (Barber, 2020). Furthermore, the same oxidation states of the four Mn ions in **4**, **7**, **8,** and **9** (Figures 5, 7, 9) further confirm the assignment of the oxidation stats of (III, III, IV, IV) in the S_1_ state OEC in PSII (Krewald et al., 2015).

## Calcium Substituted OEC’s Mimics

The redox-inactive metal ion, Ca^2+^, is an indispensable component for the catalytic function of the OEC, and its depletion results in the complete loss of the water-splitting capability of PSII (Yocum, 2008). In the biological system, Ca^2+^ can only be functionally replaced by Sr^2+^ (Boussac et al., 2004; Yocum, 2008). It has been argued that the Lewis acidity of the redox-inactive metal ion could play a role in modulating the redox potentials of heterometallic-oxide clusters (Tsui et al., 2013b; Tsui and Agapie, 2013; Krewald et al., 2016; Saito et al., 2021). However, the detailed functional role of the Ca^2+^ in the OEC remains largely unknown because direct investigation of the calcium is severely restricted by the lack of controlled modifications of this redox-inactive metal ion without changing the core structure and the local protein environment of the OEC in the biological system (Krewald et al., 2016; Saito et al., 2021).

**Figure 10:**
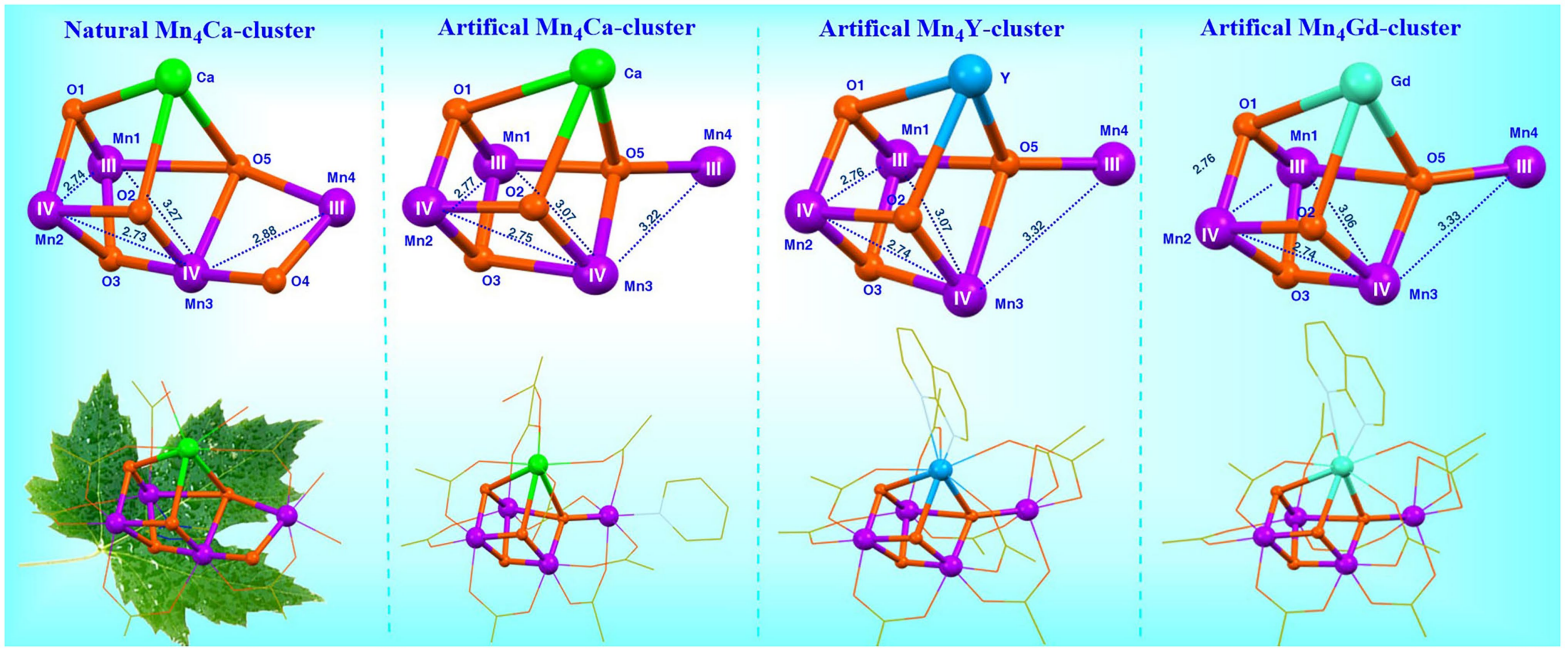
Structural comparison of the OEC (Suga et al., 2015), Mn_4_CaO_4_-cluster (**4**) (Zhang et al., 2015), the Mn_4_YO_4_-cluster (**10**) and Mn_4_GdO_4_-cluster (**11**) (Yao et al., 2021). See Figure 5 for further information.

To study the possible function of the calcium ion in OEC and to develop robust artificial catalysts for the water-splitting reaction, tremendous efforts have been devoted to preparing calcium substituted Mn_4_XO_4_-clusters in our laboratory. In 2021, we successfully prepared the [Mn_4_YO_4_(^t^BuCO_2_)_9_(Napy)] (Napy = 1,8-naphthyridine) (**10**) and [Mn_4_GdO_4_(^t^BuCO_2_)_9_(Napy)] (**11**) (Yao et al., 2021). Surprisingly, as shown in Figure 10, both the two rare-earth element-containing Mn_4_XO_4_-clusters (X = Y, Gd) have nearly the same core structure and peripheral carboxylic ligands, as well as the oxidation states of the four Mn ions as those in the Mn_4_CaO_4_-cluster (**4**) and in the S_1_ state of the OEC (Umena et al., 2011). This observation clearly demonstrates that the substitution of the calcium by the rare-earth element does not affect neither the geometric structure nor the electronic structure of the Mn_4_XO_4_-clusters.

**Figure 11:**
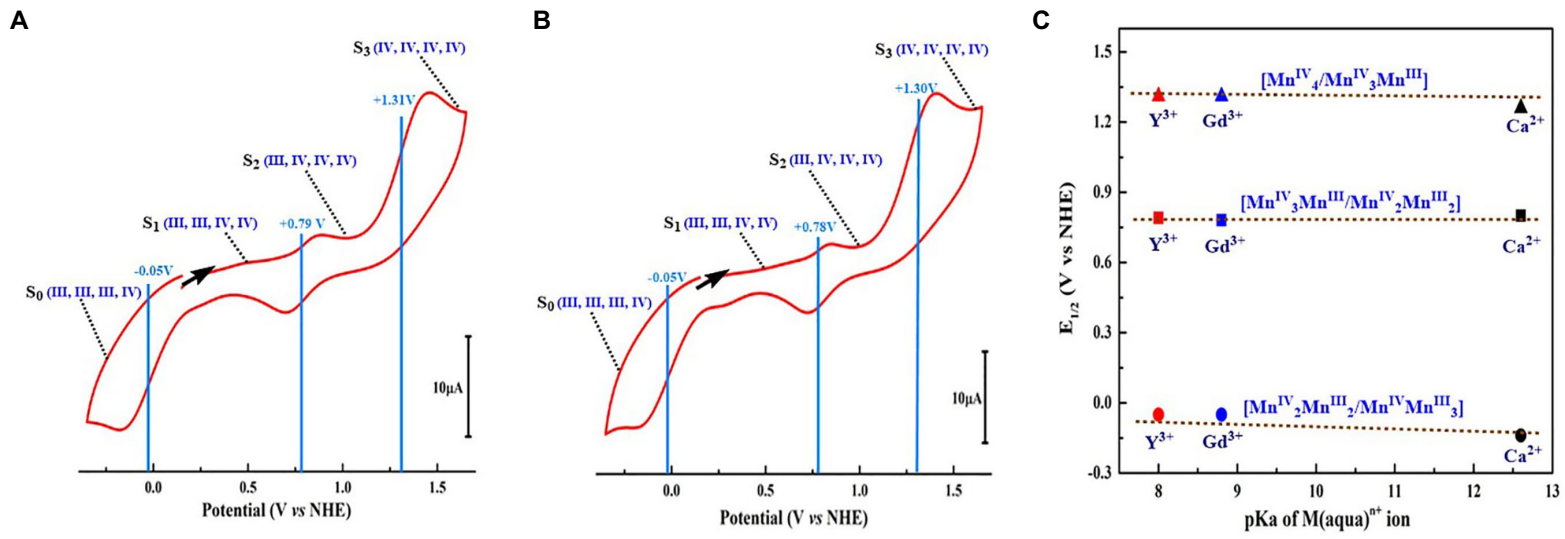
Redox potentials of the Mn_4_YO_4_-cluster (**10**) and Mn_4_GdO_4_-cluster (**11**) (Yao et al., 2021). **(A)** CV of Mn_4_YO_4_-cluster. **(B)** CV of Mn_4_GdO_4_-cluster. The possible oxidation states of the four Mn ions are shown in blue. The scan direction is indicated by the arrow. **(C)** Dependence of E1/2 of three redox couples on pKa of the X(aqua)^n+^ ion (Perrin, 1982) of three Mn4XO4-clusters. All potentials were referenced to NHE.

CV measurements (Figure 11) show that both Mn_4_YO_4_-cluster (**10**) and Mn_4_GdO_4_-cluster (**11**) have a redox potential of +0.79 V for the [Mn^III^_2_Mn^IV^_2_]/[Mn^III^Mn^IV^_3_] redox couple, which is nearly the same as that of the Mn_4_CaO_4_-cluster (**4**) (+0.8 V) (Zhang et al., 2015) and the estimated value for the S_1_ → S_2_ transition (∼ + 0.8 V) of the OEC (Dau and Zaharieva, 2009; Mandal et al., 2020). Moreover, the redox potentials of −0.05 V and + 1.3 V for the [Mn^III^_3_Mn^IV^]/[Mn^III^_2_Mn^IV^_2_] and [Mn^III^Mn^IV^_3_]/[Mn^IV^_4_] irreversible redox couples can be estimated for both the Mn_4_YO_4_-cluster and the Mn_4_GdO_4_-cluster, respectively. These values are also close to that (−0.1 and + 1.25 V) observed in the Mn_4_CaO_4_-cluster (Zhang et al., 2015) as well. These results clearly show that the replacement of the calcium by rare-earth element does not significantly affect redox potentials of the heterometallic-oxide Mn_4_XO_4_-cluster although the Lewis acidity of Y^3+^, Gd^3+^, and Ca^2+^ is significantly different. This observation challenges the earlier view that the redox-inactive metal ion would modulate the redox potentials of the heterometallic-oxide cluster (Tsui et al., 2013b; Tsui and Agapie, 2013).

The above results suggest that rare-earth elements can structurally and energetically replace the calcium in artificial neutral Mn_4_XO_4_ clusters in a chemical system, which, indeed, sheds new light on the functional role of the calcium in the OEC and supports the idea that the redox-inactive metal ion could indeed play roles in maintaining the cluster’s integrity and stability instead of modulating the redox potential of the OEC. Obviously, these robust rare-earth element-containing Mn_4_XO_4_-clusters provide a structurally well-defined molecular platform to investigate the structure–function relationship of its biological paradigm and shed new light on the design of efficient water-splitting catalysts in artificial photosynthesis.

## Challenge for Future Mimicking

Although these Mn_4_XO_4_-clusters (X = Ca/Y/Gd) closely mimic the OEC in many aspects and provide new insights into the structure–function relationship of the biological catalyst, it remains a great challenge to overcome in order to discover a precise mimic of the structure and function of the OEC in a laboratory. In the first place, the μ_2_-oxide bridge (O4) seen in the S_1_ state of the OEC is still missing in all the current known Mn_4_XO_4_-clusters. Incorporating this last “missing puzzle” into artificial Mn_4_XO_4_-cluster is a great challenge for synthetic chemistry, which is urgently needed for the understanding of the functional role of this oxide bridge and of the catalytic mechanism for the O=O bond formation in the OEC. Further, all synthetic Mn_4_XO_4_-clusters display very poor solubility in aqueous solution because of the hydrophobic peripheral environment mainly provided by the pivalate groups (i. e. ^t^BuCO_2_); thus, it is difficult to carry out its catalytic performance in aqueous solution as is the case with many other artificial catalysts reported thus far (Zhang and Sun, 2019b; Kondo et al., 2021). In addition, it has been found that many Mn complexes are not stable in the aqueous solution during the catalytic reaction (Hocking et al., 2011; Li et al., 2017); thus, it is crucial to develop a proper experimental condition for the catalytic performance of these OEC’s mimics. In the biological system, the Mn_4_CaO_5_-cluster is surrounded by non-aqueous protein environment with special channels for the delivery of protons, electrons, and the substrate (Shen, 2015; Hussein et al., 2021). Obviously, mimicking the first and the second coordination spheres of the OEC in PSII with functional channels is further required to achieve high reactivity in the future.

## Concluding Remarks

In summary, the crystallographic studies of PSII have revealed that the OEC is composed of an asymmetric Mn_4_CaO_5_-cluster; however, the detailed catalytic mechanism for the water-splitting reaction remains elusive due to the structural uncertainty of the different intermediate states of the OEC during its catalytic turnover. It is a great challenge to precisely mimic the OEC in the laboratory, yet a series of artificial Mn_4_XO_4_-clusters (X = Ca/Y/Gd) have been reported recently, which closely mimic both the geometric structure and the electronic structure, as well as the redox properties of the OEC in PSII. The investigation of these structurally well-defined chemical models provides distinct chemical insights into the understanding of the structure–function relationship of the OEC as well as the catalytic mechanism of the water-splitting reaction in natural photosynthesis. We list below several major take-home messages.

The oxidation states of the four Mn ions in all these Mn_4_XO_4_-clusters are (III, III, IV, IV), which provides the unambiguous chemical evidence for the “high-oxidation paradigm” assignment of the four Mn ions in the S_1_ state of the OEC (Krewald et al., 2015).

The preparation and reactivity of artificial Mn_4_CaO_4_-clusters clearly demonstrate that this cluster is thermodynamically stable, which supports the proposal that the Mn_4_CaO_4_-cluster could be an evolutionary origin of the natural OEC (Barber, 2016).

The finding that the rare-earth elements can structurally and energetically replace the calcium in the Mn_4_CaO_4_-cluster provides important chemical insight into the functional role of the calcium in the OEC. It indicates that the redox-inactive metal ion could play roles in maintaining the cluster’s integrity and stability instead of modulating the redox potential of the OEC.

Based on the characterization of artificial Mn_4_XO_4_-clusters, we clearly see that all μ_3_- and μ_4_- oxide bridges are tightly bound to the cluster, supporting that they may play roles in maintaining the cluster’s stability and integrity rather than as reactive sites for the O=O bond formation. However, we should point out that precise structural mimicking and functional mimicking are urgently required in the future to reveal the detailed catalytic mechanism and to achieve the high reactivity of water-splitting reaction in artificial photosynthesis. We believe that the further investigation of these robust artificial Mn_4_XO_4_-clusters would help to develop efficient man-made catalysts for the water-splitting reaction in artificial photosynthesis.

## Author Contributions

CZ conceived the project and designed experiments. YC carried out BVS calculation. CZ, CC, BX, and RY participated in the synthesis and characterization of artificial Mn_4_XO_4_-clusters involved in the paper. YC and CZ wrote the manuscript All authors contributed to the article and approved the submitted version.

## Funding

This work was supported by the National Natural Science Foundation of China (Nos. 91961203 and 22001255), the National Key Research & Development Program of China (No. 2017YFA0503704), the Strategic Priority Research Program of the Chinese Academy of Sciences (Nos. XDA21010212 and XDB17030600), and the Youth Innovation Promotion Association CAS (No. 2022030).

## Conflict of Interest

The authors declare that the research was conducted in the absence of any commercial or financial relationships that could be construed as a potential conflict of interest.

## Publisher’s Note

All claims expressed in this article are solely those of the authors and do not necessarily represent those of their affiliated organizations, or those of the publisher, the editors and the reviewers. Any product that may be evaluated in this article, or claim that may be made by its manufacturer, is not guaranteed or endorsed by the publisher.
